# Supplementary material for: From Metabolic Syndrome to Cardio-Kidney-Metabolic Syndrome in the SIMETAP Study: Prevalence Rates of Metabolic Syndrome and Its Independent Associations with Cardio-Renal-Metabolic Disorders Other than Its Defining Criteria
Source: Biomedicines. 2025 Feb 28;13(3):590. doi: 10.3390/biomedicines13030590 (PMC11939902; doi:10.3390/biomedicines13030590)
Supplement: Supplementary file 1 [file biomedicines-13-00590-s001.zip › biomedicines-3467418-supplementary.pdf]

From Metabolic Syndrome to Cardio-Kidney-Metabolic Syndrome in the SIMETAP Study: Prevalence Rates of Metabolic Syndrome and its Independent Associations with Cardio-Renal-Metabolic Disorders other than its Defining Criteria

SUPPLEMENTARY MATERIALS

Table S1. Definitions of diseases and clinical conditions criteria

Figure S1: Age-specific prevalence rates of MetS according to NCEP/ATP-III

Figure S2: Age-specific prevalence rates of MetS according to IDF

Table S2. Diseases and medical conditions in populations with and without MetS

Table S3. Diseases and medical conditions in populations with morbid and premorbid MetS

Table S4. Diseases and medical conditions in populations with morbid MetS and without MetS

Table S5. Diseases and medical conditions in populations with premorbid MetS and without MetS

Table S6. Multivariate analysis of diseases and medical conditions for MetS (a), for morbid MetS (b), and for premorbid MetS (c)

Table S1. Definitions of diseases and clinical conditions criteria

**Morbidities, diseases or clinical conditions**	**Definitions and criteria**
Current smoking	Any amount of tobacco use in the previous year.
Alcoholism	> 21 standard drink units (SDU) of alcohol consumption per week (male), > 14 SDU per week (female).<br>1 SDU is equivalent to 10 g of alcohol.
Physical inactivity [49]	Lack of moderate-intensity physical activity (e.g., brisk walking) < 150 minutes a week, or vigorous-intensity physical activity (e.g., jogging) < 75 minutes a week (or less of equivalent combination of both), or muscle strengthening exercises < 2 days a week.
Body mass index (BMI)	BMI was calculated as weight in kilograms divided by the square of height in meters.
Overweight [50]	BMI 25.0–29.9 kg/m^2 (International Classification of Diseases, 10th Revision, Clinical Modification [ICD-10-CM]: E66.3; International Classification of Primary Care, 2nd edition [ICPC-2]: T83)*
Obesity [50]	BMI ≥ 30 kg/m^2 (ICD-10-CM: E66.9; ICPC-2: T82)*
Waist circumference (WC)	WC determined with the subject standing using a flexible tape measure adjusted without compressing the skin, after the participant exhaled a normal breath, locating the upper edge of the iliac crests and above that point surrounding the waist parallel to the floor.
Abdominal (or central) obesity (AO) [13]	WC ≥ 102 cm in men or ≥ 88 cm in women
Waist-to-height ratio (WtHR)	WtHR is calculated as WC measurement divided by height measurement, both in centimetres.
High-WtHR [51]	WtHR ≥ 0.60 for both male and female populations.
CUN-BAE adiposity [52]	CUN-BAE (according to its acronym in Spanish, *Clínica Universitaria de Navarra* - Body Adiposity Estimator) body fat index:<br>• Male: −44.988 + (0.503 x age) + (3.172 x BMI) − (0.026 x BMI^2) − (0.02 x BMI x age) + (0.00021 x BMI^2 x age)<br>• Female: −44.988 + (0.503 x age) + 10.689 + (3.172 x BMI) − (0.026 x BMI^2) + (0.181 x BMI) − (0.02 x BMI x age) − (0.005 x BMI^2) + (0.00021 x BMI^2 x age)
CUN-BAE excess adiposity (EA) [52]	CUN-BAE body fat index > 25% for men or > 35% for women

Arterial hypertension (HTN) [53]	Systolic blood pressure (SBP) ≥ 140 mmHg and/or diastolic blood pressure (DBP) ≥ 90 mmHg, using the average of two or more readings obtained on two or more occasions, or being on blood pressure-lowering drug therapy (BPLT) (ICD-10-CM: I10, I15; ICPC-2: K86, K87)*
Pulse pressure	SBP – DBP (mmHg)
Diabetes mellitus (DM) [54]	It was defined according to the American Diabetes Association (ADA) criteria: fasting plasma glucose (FPG) ≥ 126 mg/dL (7.0 mmol/L) or glycated haemoglobin A_{1c} (HbA_{1c}) ≥ 6.5 % (in International Federation of Clinical Chemistry and Laboratory Medicine [IFCC] units) (≥ 48 mmol/mol) or plasma glucose ≥ 200 mg/dL (11.1 mmol/L) at any time or with oral glucose tolerance test (ICD-10-CM: E10, E11; ICPC-2: T89, T90)*<br>To convert from mg/dL to mmol/L, multiply by 0.05556<br>To convert from % (DCCT) to mmol/mol (IFCC), subtract 2.15 and multiply by 10.929
Prediabetes [54]	It was defined according to the ADA criteria: FPG between 100 and 125 mg/dL or HbA_{1c} between 5.7% and 6.4% (ICD-10-CM: R73.09; ICPC-2: A91)*<br>To convert from mg/dL to mmol/L, multiply by 0.05556<br>To convert from % (DCCT) to mmol/mol (IFCC), subtract 2.15 and multiply by 10.929
Estimated average glucose (eAG)	28.7 x HbA_{1c} – 46.7 (mg/dL)<br>To convert from mg/dL to mmol/L, multiply by 0.05556
Hypercholesterolaemia	Fasting plasma total cholesterol (TC) levels ≥ 200 mg/dL (≥ 5.17 mmol/L) (ICD-10-CM: E78; ICPC-2: T93)* or being on lipid-lowering drug therapy (LLT) to achieve cholesterol targets.<br>To convert from mg/dL to mmol/L, multiply by 0.02586
Hypertriglyceridaemia (HTG)	Fasting plasma triglycerides (TG) levels ≥ 150 mg/dL (≥ 1.69 mmol/L) (ICD-10-CM: E78; ICPC-2: T93)* or being on triglycerides lowering specific drug therapy.<br>To convert from mg/dL to mmol/L, multiply by 0.01129
Low high-density lipoprotein cholesterol (HDL-c)	HDL-c < 40 mg/dL (< 1.03 mmol/L) (for men)<br>HDL-c < 50 mg/dL (< 1.29 mmol/L) (for women)<br>To convert from mg/dL to mmol/L, multiply by 0.02586
Atherogenic dyslipidaemia	HTG and low HDL-c
Non-high-density lipoprotein cholesterol (Non-HDL-c)	TC – HDL-C<br>To convert from mg/dL to mmol/L, multiply by 0.02586
Low-density lipoprotein cholesterol (LDL-c)	TC – HDL-c – (TG/5) mg/dL (not valid for patients with TG > 400 mg/dL)<br>TC – HDL-c – (TG/2.2) mmol/L (not valid for patients with TG > 4.51 mmol/L)<br>LDL-c, TC, HDL-c: To convert from mg/dL to mmol/L, multiply by 0.02586<br>TG: To convert from mg/dL to mmol/L, multiply by 0.01129

Residual cholesterol (RC)	Very low-density lipoproteins cholesterol (VLDL-c) and remnants.<br>RC= TC – HDL-c – LDL-c<br>LDL-c, TC, HDL-c: To convert from mg/dL to mmol/L, multiply by 0.02586
Atherogenic index of plasma (AIP) [55]	AIP = log (TG / HDL-c)<br>TG and HDL-c are expressed in mmol/L<br>AIP values between −0.3 to 0.1 are associated with a low CVD risk<br>AIP values between > 0.1 and 0.24 are associated with a medium CVD risk<br>AIP values > 0.24 are considered high risk for CVD [8]
Triglyceride-glucose (TyG) index [56]	TyG index = Ln (TG x FPG/2)<br>TG and FPG are expressed in mg/dL<br>High TyG cut-off points for detecting MetS: ≥ 8.77 for men; ≥ 8.70 for women [10]
Lipid accumulation product (LAP) [57,58]	LAP = (WC − 65) x TG for men<br>LAP = (WC − 58) x TG for women<br>WC is expressed in cm, and TG in mmol/L<br>High VAI cut-off points: ≥ 59.85 for men; ≥ 53.06 for women [10]
Visceral adiposity index (VAI) [59]	VAI = {WC/[39.68 + (1.88 × BMI)]} × (TG/1.03) × (1.31/HDL-c) for men<br>VAI = {WC/[36.58 + (1.89 × BMI)]} × (TG/0.81) × (1.52/HDL-c) for women<br>WC is expressed in cm, BMI in kg/m^2, TG and HDL-c in mmol/L.<br>High VAI cut-off points for detecting MetS: ≥ 2.36 for men; ≥ 2.92 for women [10]
Metabolic syndrome (MetS) according to 2009 Joint Statement (2009-JS) [13]	MetS according to 2009 Joint Statement of International Diabetes Federation Task Force on Epidemiology and Prevention, National Heart, Lung, and Blood Institute, American Heart Association, World Heart Federation, International Atherosclerosis Society, and International Association for the Study of Obesity [3].<br>MetS diagnosis is identify by the presence of three or more of the following criteria for the European population:<br>• Increased WC (≥ 102 cm for men; ≥ 88 cm for women)<br>• TG ≥ 150 mg/dL (≥ 1.7 mmol/L)<br>• HDL-c < 40 mg/dL (< 1.03 mmol/L) (males); < 50 mg/dL (< 1.29 mmol/L) (females)<br>• SBP ≥ 130 mmHg or DBP ≥ 85 mmHg (antihypertensive drug treatment in a patient with a history of HTN is an alternate indicator)<br>• FPG ≥ 100 mg/dL (≥ 5.6 mmol/L) (drug treatment for elevated glucose is an alternate indicator)
Premorbid MetS	People with MetS and without DM or ASCVD
Morbid MetS	People with MetS and DM and/or ASCVD

MetS according to NCEP/ATP-III [10]	Subjects with MetS according to the National Cholesterol Education Program Adult Treatment Panel III report (NCEP/ATP-III) [8] must have three or more of the following criteria:<br>• Increased WC (> 102 cm for men; > 88 cm for women)<br>• TG ≥ 150 mg/dL (≥ 1.7 mmol/L)<br>• HDL-c < 40 mg/dL (< 1.03 mmol/L) (males); < 50 mg/dL (< 1.29 mmol/L) (females)<br>• SBP ≥ 130 mmHg or DBP ≥ 85 mmHg<br>• FPG ≥ 110 mg/dL (≥ 6.1 mmol/L)
MetS according to IDF [12]	Subjects with MetS according to the International Diabetes Federation (IDF) [9] must have:<br>• Central obesity (defined as increased WC for Europids): ≥ 94 cm for men; ≥ 80 cm for women.<br>plus any two of the following four factors:<br>• TG ≥ 150 mg/dL (≥ 1.7 mmol/L) or specific treatment for this lipid abnormality<br>• HDL-c < 40 mg/dL (< 1.03 mmol/L) in males; < 50 mg/dL (< 1.29 mmol/L) in females, or specific treatment for this lipid abnormality<br>• SBP ≥ 130 mmHg or DBP ≥ 85 mmHg or treatment of previously diagnosed HTN<br>• FPG ≥ 100 mg/dL (≥ 5.6 mmol/L) or previously diagnosed type 2 diabetes
Fatty liver index (FLI) [33]	FLI = $(e^{0.953*\log_e(TG) + 0.139 \times BMI + 0.718 \times \log_e(GGT) + 0.053 \times \text{waist circumference} - 15.745}) / (1 + e^{0.953*\log_e(TG) + 0.139 \times BMI + 0.718 \times \log_e(GGT) + 0.053 \times \text{waist circumference} - 15.745}) \times 100$<br>$\log_e$ = natural logarithm; GGT = gamma-glutamyl-transferase<br>A value FLI between 0 and 30 can be used to rule out steatotic liver disease (SLD) (sensitivity: 87%; negative likelihood ratio: 0.2)<br>A value FLI between 60 and 100 can be used to rule in SLD (specificity 86%; positive likelihood ratio: 4.3).
Hyperuricaemia (HU) [60]	Serum uric acid (SUA) levels ≥ 7.0 mg/dL (416 µmol/L) for both adult male and female populations, or being on urate-lowering therapy (ULT) (ICD-10-CM: E79; ICPC-2: T92)*<br>To convert from mg/dL to mmol/L, multiply by 0.05948
Coronary heart disease (CHD)	Ischemic heart disease, acute myocardial infarction, acute coronary syndrome, coronary revascularization (ICD-10-CM: I20-I25; ICPC-2: K74, K75, K76)*
Cerebrovascular disease (stroke)	Cerebral ischemia, intracranial haemorrhage, transient ischemic attack (ICD-10-CM: I60-I66, I66, I67; ICPC-2: K89, K90K K91)*
Peripheral arterial disease (PAD)	Intermittent claudication, ankle-brachial index ≤ 0.9 (ICD-10-CM: I70.2, I73.9; ICPC-2: K92)*
Atherosclerotic cardiovascular disease (ASCVD)	ASCVD include CHD, stroke, or PAD (ICD-10-CM: I70)*

Heart failure (HF) [61]	Record of HF diagnosis (ICD-10-CM: I50; ICPC-2: K77)* in the primary care electronic health records, without differentiating by phenotype based on measurement of left ventricular ejection fraction or based on severity of symptoms and physical activity.
Atrial fibrillation (AF) [62,63]	Record of AF diagnosis (ICD-10-CM: I48; ICPC-2: K78)* in the primary care electronic health records, without differentiating by phenotypes based on paroxysmal, persistent, long-standing persistent, or permanent AF or atrial flutter.
Estimated glomerular filtration rate (eGFR) [64]	According to Chronic Kidney Disease Epidemiology Collaboration (CKD-EPI) equations:<br>Women with creatinine ≤ 0.7 mg/dL= 144 x $(creatinine)^{-0.329}$ x $(0.993)^{age}$ mL/min/1.73 m^2 of the body surface<br>Women with creatinine > 0.7 mg/dL= 144 x $(creatinine)^{-1.209}$ x $(0.993)^{age}$ mL/min/1.73 m^2 of the body surface<br>Men with creatinine ≤ 0.9 mg/dL= 141 x $(creatinine)^{-0.411}$ x $(0.993)^{age}$ mL/min/1.73 m^2 of the body surface<br>Men with creatinine > 0.9 mg/dL= 141 x $(creatinine)^{-1.209}$ x $(0.993)^{age}$ mL/min/1.73 m^2 of the body surface
Low eGFR [65]	eGFR < 60 mL/min/1.73 m^2 according to CKD-EPI [20]<br>Low eGFR does not include the following categories:<br>• G1: ≥ 90 mL/min/1.73 m^2<br>• G2: 60 to 89 mL/min/1.73 m^2<br>Low eGFR includes the following categories:<br>• G3a: 45 to 59 mL/min/1.73 m^2<br>• G3b: 30 to 44 mL/min/1.73 m^2<br>• G4: 15 to 29 mL/min/1.73 m^2<br>• G5: < 15 mL/min/1.73 m^2
Albuminuria [65]	Urine albumin-creatinine ratio (uACR) ≥ 30 mg/g (including proteinuria [uACR > 300 mg/g] (ICD-10-CM: R80; ICPC-2: U98)*<br>Albuminuria does not include the following category:<br>• A1: < 30 mg/g<br>Albuminuria includes the following categories:<br>• A2: 30 mg/g to 300 mg/g<br>• A3: > 300 mg/g<br>To convert from mg/g to mg/mmol, multiply by 0.01131
Chronic kidney disease (CKD) [65]	Low eGFR and/or albuminuria (ICD-10-CM: N18; ICPC-2: U99)*
CKD risk categories [65]	Risk of CKD progression, acute kidney injury, kidney failure replacement therapy, all-cause mortality and cardiovascular events.<br>• Low risk: G1A1; G2A1<br>• Moderate risk: G1A2; G2A2; G3aA1<br>• High risk: G1A3; G2A3; G3aA2; G3bA1<br>• Very high risk: G3aA3; G3bA2; G3bA3; G4A1; G4A2; G4A3; G5A1; G5A2; G5A3

Cardiovascular risk (CVR) categories [66]	Ten-year fatal and non-fatal cardiovascular disease (CVD) risk for patients from low-risk European countries.<br>Low, moderate, high and very high CVR categories were estimated according to 2021 ESC Guidelines on cardiovascular disease prevention in clinical practice [20].
Cardiovascular-kidney-metabolic (CKM) syndrome [15]	Systemic disorder attributable to pathophysiological interactions among metabolic risk factors, CKD, and CVD, that includes both individuals at risk for CVD, CKD, and those with existing clinical CVD.<br>• Stage 0: BMI < 25 kg/m^2, normal abdominal circumference (< 88 in women and < 102 cm in men) without criteria for the other stages.<br>• Stage 1: adiposity excess (CUN-BAE obesity), overweight, obesity, abdominal obesity, or prediabetes.<br>• Stage 2: HTN, metabolic risk factors (HTG, DM, MetS), CKD moderate or high risk.<br>• Stage 3: subclinical target organ damage, risk equivalents (high CVR or CKD very high-risk) among individuals with stages 1 or 2.<br>• Stage 4: clinical CVD including CHD, stroke, PAD, HF, and AF among individuals with stages 1 or 2 (stage 4a: without CKD; stage 4b: with CKD).<br>Stages 3 or 4 are defined as advanced stages of CKM syndrome because they identify individuals at high risk for CVD.

ICD-10CM and ICPC-2 codes:

* National Center for Health Statistics (NCHS). International Classification of Diseases, Tenth Revision, Clinical Modification (ICD-10-CM). Available online: https://www.cdc.gov/nchs/icd/icd-10-cm/index.html. (accessed January 21, 2025).

* World Health Organization. (2009). International Classification of Primary Care, 2nd edition -ICPC-2. Available online: https://www.who.int/standards/classifications/other-classifications/international-classification-of-primary-care. (accessed January 21, 2025).

Figure S1: Age-specific prevalence rates of MetS according to NCEP/ATP-III

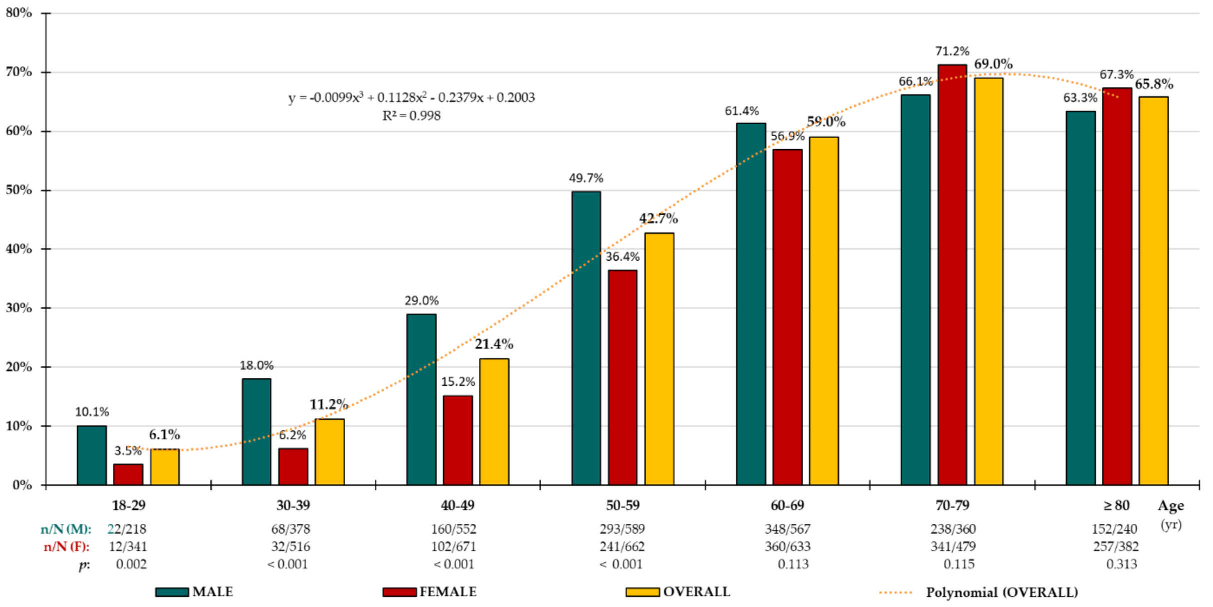

MetS: metabolic syndrome according to National Cholesterol Education Program's Adult -Treatment Panel III report (NCEP/ATP-III) [10]
n: number of cases; N: sample size; M: male; F: female; *p*: *p*-value of the difference in percentages (M *vs.* F)

Figure S2: Age-specific prevalence rates of MetS according to IDF

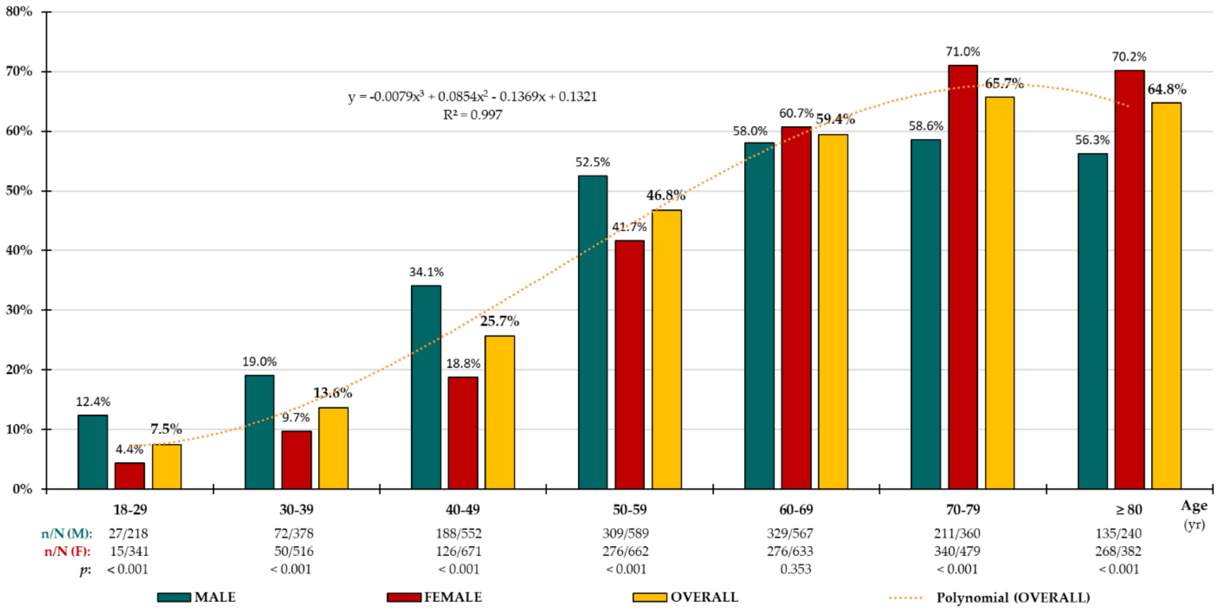

MetS: metabolic syndrome according to International Diabetes Federation (IDF) [12]
n: number of cases; N: sample size; M: male; F: female; *p*: *p*-value of the difference in percentages (M *vs.* F)

Table S2. Diseases and medical conditions in populations with and without MetS

	With MetS N= 2851 No. (%)	Without MetS N= 3737 No. (%)	With MetS *vs.* without MetS	
			p	OR (95% CI)
Current smoking	545 (19.1)	881 (23.6)	< 0.001	0.4 (0.4–0.5)
Alcoholism	305 (10.7)	305 (8.2)	< 0.001	1.3 (1.1–1.6)
Physical inactivity	1440 (50.5)	1639 (43.9)	< 0.001	1.3 (1.2–1.4)
Overweight	1165 (40.9)	1351 (36.2)	< 0.001	1.2 (1.1–1.3)
Obesity	1322 (57.3)	511 (13.7)	< 0.001	5.5 (4.8–6.1)
Abdominal obesity	2036 (71.4)	886 (23.7)	< 0.001	8.0 (7.2–9.0)
WtHR ≥ 0.60	1748 (61.3)	613 (16.4)	< 0.001	8.1 (7.2–9.1)
CUN-BAE excess adiposity	2720 (95.4)	2112 (56.5)	< 0.001	16.0 (13.3–19.3)
High VAI	895 (31.4)	221 (5.9)	< 0.001	7.3 (6.2–8.5)
Prediabetes	1042 (36.5)	407 (10.9)	< 0.001	4.7 (4.1–5.4)
Diabetes	928 (32.6)	108 (2.9)	< 0.001	16.2 (13.2–19.9)
Hypertension	2033 (71.3)	653 (17.5)	< 0.001	11.7 (10.4–13.2)
Hypercholesterolaemia	2394 (84.0)	1738 (46.5)	< 0.001	6.0 (5.3–6.8)
Low HDL-C	1289 (45.2)	530 (14.2)	< 0.001	5.0 (4.4–5.6)
Hypertriglyceridaemia	1490 (52.3)	457 (12.2)	< 0.001	7.9 (7.0–8.9)
Atherogenic dyslipidaemia	831 (29.1)	110 (2.9)	< 0.001	13.6 (11.0–16.7)
AIP > 0.24	770 (27.0)	206 (5.5)	< 0.001	6.3 (5.4–7.5)
High LAP index	1441 (50.5)	370 (9.9)	< 0.001	9.3 (8.2–10.6)
High TyG index	1560 (54.7)	512 (13.7)	< 0.001	7.6 (6.8–8.6)
FLI ≥ 60 [a]	1599 (60.2)	551 (16.0)	< 0.001	8.0 (7.1–9.0)
Coronary heart disease	296 (10.4)	25 (0.7)	< 0.001	17.2 (11.4–26.0)
Stroke	202 (7.1)	48 (1.3)	< 0.001	5.9 (4.3–8.1)
Peripheral arterial disease	127 (4.5)	23 (0.6)	< 0.001	7.5 (4.8–11.8)
ASCVD	526 (18.4)	89 (2.4)	< 0.001	9.3 (7.4–11.7)
Heart failure	151 (5.3)	33 (0.9)	< 0.001	6.3 (4.3–9.2)
Atrial fibrillation	197 (6.9)	53 (1.4)	< 0.001	5.2 (3.8–7.0)
Cardiovascular diseases	684 (24.0)	147 (3.9)	< 0.001	7.7 (6.4–9.3)
Erectile dysfunction [b]	376 (27.0)	128 (8.5)	< 0.001	4.0 (3.2–5.0)
Hyperuricaemia [c]	493 (17.5)	247 (6.7)	< 0.001	2.9 (2.5–3.5)
Diabetes or ASCVD	1222 (42.9)	190 (5.1)	< 0.001	14.0 (11.9–16.5)
Diabetes and ASCVD	232 (8.1)	7 (0.2)	< 0.001	47.2 (22.2–100.3)
GLT	771 (27.0)	76 (2.0)	< 0.001	17.9 (14.0–22.7)
LLT	1726 (60.5)	126 (3.4)	< 0.001	44.0 (36.3–53.3)
BPLT	1826 (64.0)	513 (13.7)	< 0.001	11.2 (9.9–12.6)
ULT	105 (3.7)	31 (0.8)	< 0.001	4.6 (3.0–6.8)
Albuminuria	292 (10.2)	102 (2.7)	< 0.001	4.1 (3.2–5.1)
Low eGFR	393 (13.8)	131 (3.5)	< 0.001	4.4 (3.6–5.4)
CKD	560 (19.6)	196 (5.2)	< 0.001	4.4 (3.7–5.2)
CKD low risk	2291 (80.4)	3541 (94.8)	< 0.001	0.2 (0.2–0.3)
CKD moderate risk	346 (12.1)	144 (3.9)	< 0.001	3.4 (2.8–4.2)
CKD high risk	131 (4.6)	33 (0.9)	< 0.001	5.4 (3.7–7.9)
CKD very high risk	83 (2.9)	19 (0.5)	< 0.001	5.9 (3.6–9.7)
Moderate CVR	591 (20.7)	788 (21.1)	0.729	1.0 (0.9–1.1)
High CVR	661 (23.2)	362 (9.7)	< 0.001	2.8 (2.4–3.2)
Very high CVR	1599 (56.1)	442 (11.8)	< 0.001	9.5 (8.4–10.8)

No. (%): cases number (percentage); CI: confidence interval; OR: odds ratio; *p*: *p*-value of the difference in percentage

[a] N= 2657 with MetS, 3451 without MetS; [b] N= 1392 with MetS, 1512 without MetS; [c] N= 2814 with MetS, 3675 without MetS

AIP: atherogenic index of plasma; ASCVD: atherosclerotic cardiovascular disease; BPLT: blood pressure-lowering drug therapy; CKD: chronic kidney disease; CUN-BAE: according to its acronym in Spanish, *Clínica Universitaria de Navarra* - Body Adiposity Estimator; CVR: cardiovascular risk; eGFR: estimated glomerular filtration rate; FLI: fatty liver index; GLT: glycaemic-lowering drug therapy; HDL-C: high-density lipoprotein cholesterol; LAP: lipid accumulation product index; LLT: lipid-lowering drug therapy; MetS: metabolic syndrome; TyG: triglyceride-glucose index; ULT: urate-lowering drug therapy; VAI: visceral adiposity index.

The definitions of diseases or medical conditions are shown in Table S1 (Supplementary Materials).

Table S3. Diseases and medical conditions in populations with morbid and premorbid MetS

	Morbid MetS N= 1222 No. (%)	Premorbid MetS N= 1629 No. (%)	Morbid *vs.* premorbid MetS	
			p	OR (95% CI)
Current smoking	199 (16.3)	346 (21.2)	0.001	0.7 (0.6–0.9)
Alcoholism	126 (10.3)	179 (11.0)	0.563	0.9 (0.7–1.2)
Physical inactivity	596 (48.8)	844 (51.8)	0.108	0.9 (0.8–1.0)
Overweight	489 (40.0)	676 (41.5)	0.426	0.9 (0.8–1.1)
Obesity	568 (46.5)	754 (46.3)	0.918	1.0 (0.9–1.2)
Abdominal obesity	845 (69.1)	1191 (73.1)	0.020	0.8 (0.7–1.0)
WtHR ≥ 0.60	797 (65.2)	951 (58.4)	< 0.001	1.3 (1.1–1.6)
CUN-BAE excess adiposity	1169 (95.7)	1551 (95.2)	0.569	1.1 (0.8–1.6)
High VAI	377 (30.9)	518 (31.8)	0.589	1.0 (0.8–1.1)
Prediabetes	162 (13.3)	880 (54.0)	< 0.001	0.1 (0.1–0.2)
Diabetes	928 (75.9)	0 (0.0)	NA	NE
Hypertension	192 (15.7)	626 (38.4)	< 0.001	0.3 (0.2–0.4)
Hypercholesterolaemia	1080 (88.4)	1314 (80.7)	< 0.001	1.8 (1.5–2.3)
Low HDL-C	564 (46.2)	725 (44.5)	0.382	1.1 (0.9–1.2)
Hypertriglyceridaemia	631 (51.6)	859 (52.7)	0.562	1.0 (0.8–1.1)
Atherogenic dyslipidaemia	369 (30.2)	462 (28.4)	0.286	1.1 (0.9–1.3)
AIP > 0.24	328 (26.8)	442 (27.1)	0.863	1.0 (0.8–1.2)
High LAP index	590 (48.3)	851 (52.2)	0.036	0.9 (0.7–1.0)
High TyG index	733 (60.0)	827 (50.8)	< 0.001	1.5 (1.3–1.7)
FLI ≥ 60 [a]	683 (59.9)	916 (60.4)	0.806	1.0 (0.8–1.1)
Coronary heart disease	296 (24.2)	0 (0.0)	NA	NE
Stroke	202 (16.5)	0 (0.0)	NA	NE
Peripheral arterial disease	127 (10.4)	0 (0.0)	NA	NE
ASCVD	526 (43.0)	0 (0.0)	NA	NE
Heart failure	109 (8.9)	42 (2.6)	< 0.001	3.7 (2.6–5.3)
Atrial fibrillation	124 (10.1)	73 (4.5)	< 0.001	2.4 (1.8–3.2)
Cardiovascular diseases	589 (48.2)	95 (5.8)	< 0.001	15.0 (11.9–19.0)
Erectile dysfunction [b]	271 (40.5)	105 (14.5)	< 0.001	4.0 (3.1–5.2)
Hyperuricaemia [c]	213 (17.7)	280 (17.4)	0.795	1.0 (0.8–1.2)
GLT	765 (62.6)	6 (0.4)	< 0.001	453 (201–1018)
LLT	933 (76.4)	793 (48.7)	< 0.001	3.4 (2.9–4.0)
BPLT	950 (77.7)	876 (53.8)	< 0.001	3.0 (2.5–3.5)
ULT	50 (4.2)	55 (3.4)	0.297	1.2 (0.8–1.8)
Albuminuria	196 (16.0)	96 (5.9)	< 0.001	3.1 (2.4–3.9)
Low eGFR	233 (19.1)	160 (9.8)	< 0.001	2.1 (1.7–2.7)
CKD	344 (28.2)	216 (13.3)	< 0.001	2.6 (2.1–3.1)
CKD low risk	878 (71.8)	1413 (86.7)	< 0.001	0.4 (0.3–0.5)
CKD moderate risk	200 (16.4)	146 (9.0)	< 0.001	2.0 (1.6–2.5)
CKD high risk	89 (7.3)	42 (2.6)	< 0.001	3.0 (2.0–4.3)
CKD very high risk	55 (4.5)	28 (1.7)	< 0.001	2.7 (1.7–4.3)
Moderate CVR	2 (0.2)	589 (36.2)	< 0.001	0.0 (0.0–0.01)
High CVR	1 (0.1)	660 (40.5)	< 0.001	0.0 (0.0–0.01)
Very high CVR	1219 (99.8)	380 (23.3)	< 0.001	1335 (428–4171)

No. (%): cases number (percentage); CI: confidence interval; OR: odds ratio; *p*: *p*-value of the difference in percentage; NA: not applicable; NE: not estimable.

[a] N= 1140 with morbid MetS, 1517 with premorbid MetS; [b] N= 669 with morbid MetS, 723 with premorbid MetS; [c] N= 1201 with morbid MetS, 1613 with premorbid MetS

AIP: atherogenic index of plasma; ASCVD: atherosclerotic cardiovascular disease; BPLT: blood pressure-lowering drug therapy; CKD: chronic kidney disease; CUN-BAE: according to its acronym in Spanish, *Clínica Universitaria de Navarra* - Body Adiposity Estimator; CVR: cardiovascular risk; eGFR: estimated glomerular filtration rate; FLI: fatty liver index; GLT: glycaemic-lowering drug therapy; HDL-C: high-density lipoprotein cholesterol; LAP: lipid accumulation product index; LLT: lipid-lowering drug therapy; MetS: metabolic syndrome; TyG: triglyceride-glucose index; ULT: urate-lowering drug therapy; VAI: visceral adiposity index; WtHR: waist-to-height ratio.
The definitions of diseases or medical conditions are shown in Table S1 (Supplementary Materials).

Table S4. Diseases and medical conditions in populations with morbid MetS and without MetS

	Morbid MetS N= 1222 No. (%)	Without MetS N= 3737 No. (%)	Morbid MetS *vs.* without MetS	
			p	OR (95% CI)
Current smoking	199 (16.3)	881 (23.6)	0.002	0.6 (0.5–0.7)
Alcoholism	126 (10.3)	305 (8.2)	0.021	1.3 (1.0–1.6)
Physical inactivity	596 (48.8)	1639 (43.9)	0.003	1.2 (1.1–1.4)
Overweight	489 (40.0)	1351 (36.2)	0.015	1.2 (1.0–1.3)
Obesity	568 (46.5)	511 (13.7)	< 0.001	5.5 (4.7–6.3)
Abdominal obesity	845 (69.1)	886 (23.7)	< 0.001	7.2 (6.3–8.3)
WtHR ≥ 0.60	797 (65.2)	613 (16.4)	< 0.001	9.6 (8.3–11.1)
CUN-BAE excess adiposity	1169 (95.7)	2112 (56.5)	< 0.001	17.0 (12.8–22.5)
High VAI	377 (30.9)	221 (5.9)	< 0.001	7.1 (5.9–8.5)
Prediabetes	162 (13.3)	407 (10.9)	0.024	1.3 (1.0–1.5)
Diabetes	928 (75.9)	108 (2.9)	< 0.001	106 (84–134)
Hypertension	192 (15.7)	653 (17.5)	0.155	0.9 (0.7–1.0)
Hypercholesterolaemia	1080 (88.4)	1738 (46.5)	< 0.001	8.7 (7.3–10.5)
Low HDL-C	564 (46.2)	530 (14.2)	< 0.001	5.2 (4.5–6.0)
Hypertriglyceridaemia	631 (51.6)	457 (12.2)	< 0.001	7.7 (6.6–8.9)
Atherogenic dyslipidaemia	369 (30.2)	110 (2.9)	< 0.001	14.3 (11.4–17.9)
AIP > 0.24	328 (26.8)	206 (5.5)	< 0.001	6.3 (5.2–7.6)
High LAP index	590 (48.3)	370 (9.9)	< 0.001	8.5 (7.3–9.9)
High TyG index	733 (60.0)	512 (13.7)	< 0.001	9.4 (8.4–10.9)
FLI ≥ 60 [a]	683 (59.9)	551 (16.0)	< 0.001	7.9 (6.8–9.1)
Coronary heart disease	296 (24.2)	25 (0.7)	< 0.001	47.5 (31.4–71.8)
Stroke	202 (16.5)	48 (1.3)	< 0.001	15.2 (11.0–21.0)
Peripheral arterial disease	127 (10.4)	23 (0.6)	< 0.001	18.7 (12.0–29.3)
ASCVD	526 (43.0)	89 (2.4)	< 0.001	31.0 (24.4–39.3)
Heart failure	109 (8.9)	33 (0.9)	< 0.001	11.0 (7.4–16.3)
Atrial fibrillation	124 (10.1)	53 (1.4)	< 0.001	7.9 (5.7–10.9)
Cardiovascular diseases	589 (48.2)	147 (3.9)	< 0.001	18.6 (18.6–27.7)
Erectile dysfunction [b]	271 (40.5)	128 (8.5)	< 0.001	7.4 (5.8–9.3)
Hyperuricaemia [c]	213 (17.7)	247 (6.7)	< 0.001	3.0 (2.5–3.6)
Diabetes and ASCVD	232 (19.0)	7 (0.2)	< 0.001	124.9 (58.7–265.7)
GLT	765 (62.6)	76 (2.0)	< 0.001	92.5 (75.2–115.4)
LLT	933 (76.4)	126 (3.4)	< 0.001	44.0 (36.3–53.3)
BPLT	950 (77.7)	513 (13.7)	< 0.001	22.0 (18.6–25.9)
ULT	50 (4.2)	31 (0.8)	< 0.001	5.2 (3.3–8.2)
Albuminuria	196 (16.0)	102 (2.7)	< 0.001	6.8 (5.3–8.7)
Low eGFR	233 (19.1)	131 (3.5)	< 0.001	6.5 (5.2–8.1)
CKD	344 (28.2)	196 (5.2)	< 0.001	7.1 (5.9–8.6)
CKD low risk	878 (71.8)	3541 (94.8)	< 0.001	0.1 (0.1–0.2)
CKD moderate risk	200 (16.4)	144 (3.9)	< 0.001	4.9 (3.9–6.1)
CKD high risk	89 (7.3)	33 (0.9)	< 0.001	8.8 (5.9–13.2)
CKD very high risk	55 (4.5)	19 (0.5)	< 0.001	9.2 (5.5–15.6)
Moderate CVR	2 (0.2)	788 (21.1)	< 0.001	0.01 (0.00–0.02)
High CVR	1 (0.1)	362 (9.7)	< 0.001	0.01 (0.00–0.05)
Very high CVR	1219 (99.8)	442 (11.8)	< 0.001	3029 (971–9446)

No. (%): cases number (percentage); CI: confidence interval; OR: odds ratio; *p*: *p*-value of the difference in percentage

[a] N= 1140 with morbid MetS, 3451 without MetS; [b] N= 669 with morbid MetS, 1512 without MetS; [c] N= 1201 with morbid MetS, 3675 without MetS

AIP: atherogenic index of plasma; ASCVD: atherosclerotic cardiovascular disease; BPLT: blood pressure-lowering drug therapy; CKD: chronic kidney disease; CUN-BAE: according to its acronym in Spanish, *Clínica Universitaria de Navarra* - Body Adiposity Estimator; CVR: cardiovascular risk; eGFR: estimated glomerular filtration rate; FLI: fatty liver index; GLT: glycaemic-lowering drug therapy; HDL-C: high-density lipoprotein cholesterol; LAP: lipid accumulation product index; LLT: lipid-lowering drug therapy; MetS: metabolic syndrome; TyG: triglyceride-glucose index; ULT: urate-lowering drug therapy; VAI: visceral adiposity index; WtHR: waist-to-height ratio.
The definitions of diseases or medical conditions are shown in Table S1 (Supplementary Materials).

Table S5. Diseases and medical conditions in populations with premorbid MetS and without MetS

	Premorbid MetS N= 1629 No. (%)	Without MetS N= 3737 No. (%)	Premorbid MetS *vs.* without MetS	
			p	OR (95% CI)
Current smoking	346 (21.2)	881 (23.6)	0.061	0.9 (0.8–1.0)
Alcoholism	179 (11.0)	305 (8.2)	< 0.001	1.4 (1.1–1.7)
Physical inactivity	844 (51.8)	1639 (43.9)	< 0.001	1.4 (1.2–1.5)
Overweight	676 (41.5)	1351 (36.2)	< 0.001	1.3 (1.1–1.4)
Obesity	754 (46.3)	511 (13.7)	< 0.001	5.4 (4.8–6.2)
Abdominal obesity	1191 (73.1)	886 (23.7)	< 0.001	8.8 (7.7–10.0)
WtHR ≥ 0.60	951 (58.4)	613 (16.4)	< 0.001	7.1 (6.3–8.2)
CUN-BAE excess adiposity	1551 (95.2)	2112 (56.5)	< 0.001	15.3 (12.1–19.4)
High VAI	518 (31.8)	221 (5.9)	< 0.001	7.4 (6.2–8.8)
Prediabetes	880 (54.0)	407 (10.9)	< 0.001	9.6 (8.3–11.1)
Diabetes	0 (0.0)	108 (2.9)	NA	NE
Hypertension	626 (38.4)	653 (17.5)	< 0.001	2.9 (2.6–3.4)
Hypercholesterolaemia	1314 (80.7)	1738 (46.5)	< 0.001	4.8 (4.2–5.5)
Low HDL-C	725 (44.5)	530 (14.2)	< 0.001	4.9 (4.2–5.5)
Hypertriglyceridaemia	859 (52.7)	457 (12.2)	< 0.001	8.0 (7.0–9.2)
Atherogenic dyslipidaemia	462 (28.4)	110 (2.9)	< 0.001	13.1 (10.5–16.2)
AIP > 0.24	442 (27.1)	206 (5.5)	< 0.001	6.4 (5.3–7.6)
High LAP index	851 (52.2)	370 (9.9)	< 0.001	10.0 (8.6–11.5)
High TyG index	827 (50.8)	512 (13.7)	< 0.001	6.5 (5.7–7.4)
FLI ≥ 60 [a,b]	916 (60.4)	551 (16.0)	< 0.001	8.0 (7.0–9.2)
Coronary heart disease	0 (0.0)	25 (0.7)	NA	NE
Stroke	0 (0.0)	48 (1.3)	NA	NE
Peripheral arterial disease	0 (0.0)	23 (0.6)	NA	NE
ASCVD	0 (0.0)	89 (2.4)	NA	NE
Heart failure	42 (2.6)	33 (0.9)	< 0.001	3.0 (1.9–4.7)
Atrial fibrillation	73 (4.5)	53 (1.4)	< 0.001	3.3 (2.3–4.7)
Cardiovascular diseases	95 (5.8)	147 (3.9)	0.002	1.5 (1.2–2.0)
Erectile dysfunction [c,d]	105 (14.5)	128 (8.5)	< 0.001	1.8 (1.4–2.4)
Hyperuricaemia [e,f]	280 (17.4)	247 (6.7)	< 0.001	2.9 (2.4–3.5)
GLT	6 (0.4)	76 (2.0)	< 0.001	0.2 (0.1–0.4)
LLT	793 (48.7)	126 (3.4)	< 0.001	27.2 (22.2–33.3)
BPLT	876 (53.8)	513 (13.7)	< 0.001	7.3 (3.4–8.4)
ULT	55 (3.4)	31 (0.8)	< 0.001	4.2 (2.7–6.6)
Albuminuria	96 (5.9)	102 (2.7)	< 0.001	2.2 (1.7–3.0)
Low eGFR	160 (9.8)	131 (3.5)	< 0.001	3.0 (2.4–3.8)
CKD	216 (13.3)	196 (5.2)	< 0.001	2.8 (2.3–3.4)
CKD low risk	1413 (86.7)	3541 (94.8)	< 0.001	0.4 (0.3–0.4)
CKD moderate risk	146 (9.0)	144 (3.9)	< 0.001	2.5 (1.9–3.1)
CKD high risk	42 (2.6)	33 (0.9)	< 0.001	3.0 (1.9–4.7)
CKD very high risk	28 (1.7)	19 (0.5)	< 0.001	3.4 (1.9–6.1)
Moderate CVR	589 (36.2)	788 (21.1)	< 0.001	2.1 (1.9–2.4)
High CVR	660 (40.5)	362 (9.7)	< 0.001	6.4 (5.5–7.4)
Very high CVR	380 (23.3)	442 (11.8)	< 0.001	2.3 (1.9–2.6)

No. (%): cases number (percentage); CI: confidence interval; OR: odds ratio; *p*: *p*-value of the difference in percentage; NA: not applicable; NE: not estimable.

[a] N= 1517 with premorbid MetS, 3451 without MetS; [b] N= 723 with premorbid MetS, 1512 without MetS; [c] N= 1613 with premorbid, MetS 3675 without MetS

AIP: atherogenic index of plasma; ASCVD: atherosclerotic cardiovascular disease; BPLT: blood pressure-lowering drug therapy; CKD: chronic kidney disease; CUN-BAE: according to its acronym in Spanish, *Clínica Universitaria de Navarra* - Body Adiposity Estimator; CVR: cardiovascular risk; eGFR: estimated glomerular filtration rate; FLI: fatty liver index; GLT: glycaemic-lowering drug therapy; HDL-C: high-density lipoprotein cholesterol; LAP: lipid accumulation product index; LLT: lipid-lowering drug therapy; MetS: metabolic syndrome; TyG: triglyceride-glucose index; ULT: urate-lowering drug therapy; VAI: visceral adiposity index; WtHR: waist-to-height ratio.
The definitions of diseases or medical conditions are shown in Table S1 (Supplementary Materials).

Table S6. Multivariate analysis of diseases and medical conditions for MetS **(a),** for morbid MetS **(b),** and for premorbid MetS **(c)**

	Wald	β [a]	OR Exp(β) [b]	*p* [c]
Diabetes	215.5	2.10 (0.14)	8.18 (6.18–10.83)	< 0.001
Hypertension	370.0	1.58 (0.08)	4.86 (4.14–5.71)	< 0.001
CUN-BAE excess adiposity	119.0	1.40 (0.13)	4.05 (3.15–5.20)	< 0.001
Hypercholesterolaemia	218.0	1.32 (0.09)	3.74 (3.14–4.46)	< 0.001
Coronary heart disease	18.8	1.28 (0.30)	3.59 (2.01–6.39)	< 0.001
High VAI	79.6	1.22 (0.14)	3.39 (2.59–4.43)	< 0.001
High TyG index	92.1	1.01 (0.11)	2.75 (2.24–3.38)	< 0.001
WtHR ≥ 0.60	94.8	1.00 (0.10)	2.71 (2.22–3.31)	< 0.001
Cardiovascular diseases	33.2	0.85 (0.15)	2.35 (1.76–3.13)	< 0.001
Obesity	15.0	0.44 (0.12)	1.56 (1.25–1.95)	< 0.001
FLI ≥ 60	6.0	0.26 (0.11)	1.30 (1.06–1.60)	0.014

(a)

	Wald	β [a]	OR Exp(β) [b]	*p* [c]
Hypertension	291.9	1.47 (0.09)	4.34 (3.67–5.14)	< 0.001
Hypercholesterolaemia	113.1	1.10 (0.10)	3.01 (2.46–3.69)	< 0.001
High TyG index	163.1	0.97 (0.08)	2.64 (2.27–3.06)	< 0.001
Heart failure	19.0	0.82 (0.19)	2.26 (1.57–3.27)	< 0.001
CUN-BAE excess adiposity	25.3	0.81 (0.16)	2.24 (1.63–3.06)	< 0.001
WtHR ≥ 0.60	58.4	0.61 (0.08)	1.84 (1.58–2.16)	< 0.001
Albuminuria	12.9	0.60 (0.17)	1.83 (1.32–2.55)	< 0.001
Atrial fibrillation	9.2	0.49 (0.16)	1.63 (1.19–2.24)	0.002
CKD	6.6	0.33 (0.13)	1.40 (1.08–1.80)	0.010

(b)

	Wald	β [a]	OR Exp(β) [b]	*p* [c]
CUN-BAE excess adiposity	130.5	1.52 (0.13)	4.59 (3.53–5.96)	< 0.001
Hypercholesterolaemia	56.2	0.59 (0.08)	1.80 (1.54–2.01)	< 0.001
Hypertension	37.2	0.42 (0.07)	1.52 (1.33–1.74)	< 0.001
High VAI	25.6	0.48 (0.09)	1.61 (1.34–1.94)	< 0.001
High LAP	25.2	0.48 (0.10)	1.61 (1.34–1.94)	< 0.001
FLI ≥ 60	12.4	0.32 (0.09)	1.37 (1.15–1.64)	< 0.001
WtHR ≥ 0.60	10.0	0.26 (0.08)	1.30 (1.10–1.52)	0.002

(c)

[a] β coefficient (± deviation); [b] OR Exp (β): odds-ratio (95% confidence interval); [c] *p*: *p*-value of Wald test with one degree of freedom. CKD: chronic kidney disease; CUN-BAE: according to its acronym in Spanish, *Clínica Universitaria de Navarra* - Body Adiposity Estimator; FLI: fatty liver index; LAP: lipid accumulation product index; MetS: metabolic syndrome; TyG: triglyceride-glucose index; VAI: visceral adiposity index; WtHR: waist-to-height ratio.

Definitions of the diseases and clinical conditions are shown in Table S1 (Supplementary Materials).
